# A Systematic Review of School-Based Nutrition Interventions for Promoting Healthy Dietary Practices and Lifestyle Among School Children and Adolescents

**DOI:** 10.7759/cureus.53127

**Published:** 2024-01-28

**Authors:** Poulomi Chatterjee, Abhay Nirgude

**Affiliations:** 1 Community Medicine, Yenepoya Medical College, Mangalore, IND

**Keywords:** health services, adolescent, children, intervention, school, malnutrition

## Abstract

Childhood malnutrition is one of the foremost community health problems in the world, particularly in developing countries like India. This current review was conducted to evaluate the effectiveness of various school-centered nutrition interventions/intervention programs developed in recent years, and their impact on the nutritional status, dietary habits, food preferences, lifestyle, and dietary behaviors in relation to diet, as well as physical activities for school children, especially adolescents. This review included studies found in the PubMed/Medline, SCOPUS, and Web of Science (WOS) databases, published from July 2017 to 2023. They were analyzed for eligibility criteria defined for this study, including school children and adolescents, school-based nutrition interventions/strategies/policies/initiatives, nutritional status, physical activity, dietary habits, and lifestyle. The Risk of Bias assessment was conducted using Review Manager version 5.4. Among 1776 potentially related studies, 108 met the eligibility criteria. Following this review, 62 studies were identified as eligible for this study, in which 38 intervention programs were discussed. A total of 13 studies were considered comprehensive and multi-component, 15 were nutrition education interventions, six were identified as physical activity interventions, and four focused on lifestyle and dietary behavior-related interventions. Another 24 of the 62 studies reviewed (approximately 39%) were either original articles, review articles, or articles pertaining to nutritional program guidelines, protocols, and/or reports. These studies uncovered a possible relationship between a decrease in BMI and school children's engagement in diet and/or physical activity. Results also suggest that these programs can be effective, although evidence for the long-term sustainability of changes in BMI was less evident and not fully substantiated/supported. Most of these findings are based on self-reported program data and may consist of biases linked to recall, selection of participants, and the desire to report favorable final measures (physical activity, lifestyle, and dietary habits). This study has the potential for use in public health programs devoted to healthy nutrition behavior and lifestyle practices. This research was primarily conducted by clinical researchers and did not receive any standardized institutional or organization-derived grant funding and support.

## Introduction and background

Malnutrition among children in India is a crucial wellbeing issue [1]. Both undernutrition and overnutrition have reached epidemic proportions in developing countries like India, where children and adolescents are most vulnerable. In these countries, wellbeing risks, especially related to malnutrition, are associated with poor and imbalanced nutrition, which are major causes of significant health concerns. These can further lead to slow cognitive and nervous system maturation [2]. Wellbeing habits and behaviors are established in early childhood and continue into adulthood [3]. In recent years, it has been observed that children's eating habits are influenced initially by family situations, with most changes occurring upon entering school, where they spend much of their time away from home [3]. Therefore, health education in schools is of utmost importance to promote adolescent health. By distributing and utilizing reliable internet information sources and encouraging critical thinking, schools can also play a significant role in helping adolescents filter out false information [4]. Obesity is a preventable disease, but with the influence of a Western lifestyle (characterized by a sedentary lifestyle and large consumption of processed foods, high-sugar beverages, sweets, fried foods, and high-fructose food items), its impact on the population is increasing dramatically. According to surveys conducted in Indian cities, the prevalence of overnutrition among school children is above 10%. Improved knowledge and behavior related to health and nutrition are highly associated with broad-level health and dietetics programs, especially in educational facilities in India [5]. The scientific literature reveals that the problem of undernutrition remains significant in school children, contributing to 22% of the country’s burden of disease [6]. The prevalence of poor nutritional status among children and adolescents greatly hinders national advancement, both socially and economically. Essential nutrition interventions have a significant effect on reducing the severity of undernutrition in India (NFHS-5). Older children and teenagers receive less attention from well-being providers compared to under-fives, especially in developing countries [7]. It is observed that at the personal level, weight loss interventions pointed at calorie input and output are frequently not fruitful in the long term. In this context, the Mid-day Meal Program, a multipurpose initiative of the Government of India, and the National Scheme known as PM Poshan (Pradhan Mantri Poshan Shakti Nirman) in schools are important policies addressing the nutritional needs of institute-going children, especially in the age group of 6-12 years, who are vulnerable to nutritional deficiencies with negative effects on growth and development [6]. The Focusing Resources on Effective School Health (FRESH) framework, developed in the early 2000s and primarily aimed at addressing undernutrition, was a pioneering international framework linking nutrition and education. Later, in 2006, the World Health Organization (WHO) launched the Nutrition Friendly School Initiative (NFSI) to address the double burden of malnutrition. These two frameworks have laid the foundation for elements in schools that promote health [7], with support from the Food Safety and Standards Authority of India (FSSAI) and the Indian Academy of Pediatrics (IAP).

The findings indicate that nutritional status encompasses political, cultural, and social dimensions, supporting the need for multidimensional actions to enhance the dietary habits and lifestyle of school-going children and adolescents [8]. The first step in developing such interventions is to reference research and create a model based on treatments that are efficient, effective, acceptable in a school context, and allow for easy outcome analysis [9].

It is critical to understand the effect of various interventions implemented at the school level to improve healthy dietary practices in the context of evolving school-based nutritional programs and policies [10]. Hence, this present study aims to contribute to the literature from a different perspective by identifying only school-based interventional strategies and their effectiveness. These are directly engaged in promoting nutritional status, nutrition-related knowledge, dietary habits, lifestyle, and physical activity among school-going children and adolescents and have not been sufficiently and particularly emphasized in other systematic review studies. The intervention programs have been selected and documented in the form of a systematic review to be used as a guide in producing strategies with the same goal, following the final selection and extraction of the data components and implementation techniques. This will assist community stakeholders in identifying gaps and undertaking further studies, as well as informing the development of intervention training programs that pursue altering dietary behaviors by endorsing better dietary habits in this populace clutch.

## Review

Methods

A systematic review has been conducted on various studies from all regions regarding school-based intervention strategies and their outcomes among school-going children and adolescents.

Selection Methods and Search Strategy

**Figure 1:**
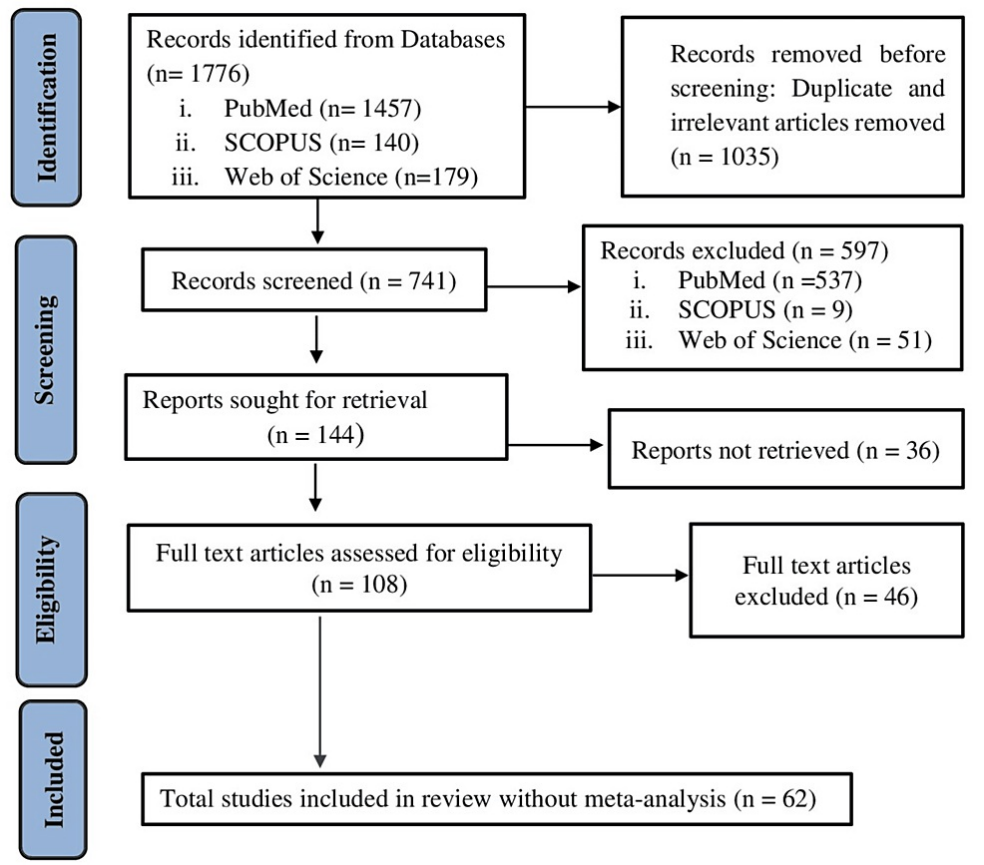
Conceptual framework and search strategy (PRISMA flow chart). PRISMA: Preferred Reporting Items for Systematic Reviews and Meta-Analyses.

By following defined enrolment criteria, autonomously all dynamic titles, abstracts, and full-texts, including additional publications, were identified from the reference lists of these articles and these have been analyzed and interpreted accordingly. A PubMed, SCOPUS, and Web of Science (WOS) exploration scheme was established and amended to additional databanks as required in June and July 2023. All these articles were screened and explored to identify nutrition-related papers by researcher one. Articles that indicated different school-based intervention strategies, such as nutrition education, health promotion, physical activity, counseling, nutrition/lifestyle management programs, etc., for school-going children and youths with outcomes clearly identifiable as nutrition-related, were reviewed by researchers one and two. When necessary, each database's approach was adjusted. In-person searches of allusion lists of previously published analyses and of included papers were conducted to ascertain additional research works. This process yielded 62 related scientific papers. The literature selection strategy is presented in Appendix 1. Figure 1 represents the search strategy in the form of a Preferred Reporting Items for Systematic Reviews and Meta-Analyses (PRISMA) flow chart.

Inclusion Criteria

Articles eligible for inclusion in this review met one or more of the following criteria: scientific literature involving school children aged six to twelve years and early and middle adolescents aged thirteen to seventeen years; studies that discussed or implemented any school-based strategy, intervention, policy, program, education, counseling, initiative, or technique; studies focusing on nutritional status, healthy eating, physical activity, dietary habits, food choices or preferences, and lifestyle; original and review articles, systematic reviews, government guidelines, protocols, or policies from July 2017 to 2023. Table 1 represents the Population Intervention Comparison Outcome (PICO) format of the inclusion criteria.

**Table 1:** PICO and other criteria for inclusion. PICO: Population Intervention Comparison Outcome.

Criterion	Inclusion
Study design	Experimental or interventional research works, Quasi-experimental studies, randomized controlled trials (RCTs) and cluster randomized controlled trials (C-RCTs), systematic reviews and review articles
Setting	School/Educational institution
Nature of publication	Scientific papers available in peer reviewed journals (original and review articles, systematic reviews, government guidelines or protocols)
Language	English
Topographical expanse	All regions
Population (P)	School children of aged 6–12 years and adolescents between 13 and 17 years
Intervention (I)	Nutrition education, diet counselling, lifestyle and behaviour, physical activity
Contrast/Controller (C)	No intervention
Outcome (O)	Improved nutrition related knowledge, dietary habits and practices, physical activity level, lifestyle and behaviour in connection to nutritional status

*Exclusion Criteria*

**Figure 2:**
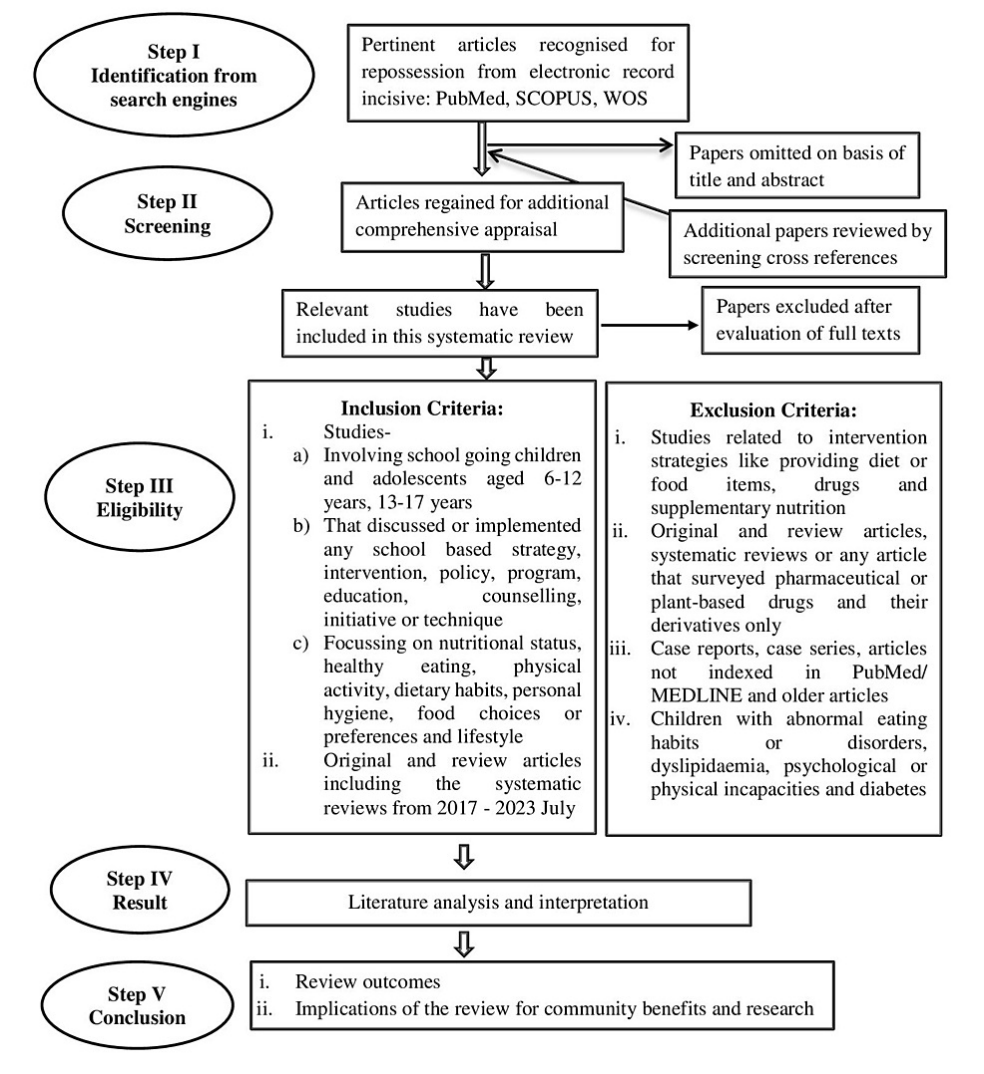
Conceptual framework of the methodology.

Articles were excluded from the review if they demonstrated one or more of the following: related to intervention strategies such as culinary activities, providing diet or food items, drugs, and supplementary nutrition; exclusively web-based, technology-based, or online; original and review articles, systematic reviews, or any articles that surveyed pharmaceutical or plant-based drugs and their derivatives only; unpublished manuscripts and conference abstracts; studies involving children with abnormal eating habits or eating disorders, dyslipidemia, psychological or physical incapacities, and diabetes. Figure 2 displays the conceptual framework of the methodology.

Data Extraction and Synthesis

A descriptive analysis was performed to extract data from each included study. The variables obtained from the selected articles were (1) Study details: author, year of publication, and country; (2) study setting and design; (3) intervention type; (4) study population: participants and their age during the intervention, along with gender; (5) sample size; (6) duration of intervention; (7) follow-up and number of dropouts; and (8) outcomes of the studies. The titles and/or abstracts and whole scripts of the relevant articles were evaluated by either author, and any inconsistencies were resolved by consensus between the authors or by both, according to the criteria for inclusion in the study. Table 2 displays the data extraction procedure.

**Table 2:** Data extraction procedure.

Criteria	Parameters
Study characteristics	Author, year of publication, country, aims and objectives of the study, participant characteristics, study design, intervention content
Study design	Randomized controlled trial, interventional or experimental, original article, systematic review, and review articles
Study population	Sample size, age with gender
Intervention characteristics	Type, duration of study, follow up
Study setting	School or educational institution
Study outcome	Improved nutrition-related knowledge, dietary habits and practices, physical activity level, lifestyle, and behavior in connection to nutritional status

Results

A total of 1776 studies were obtained from databases. Among these, 1457 studies were from PubMed, 140 from SCOPUS, and 179 from WOS. After excluding duplicate and irrelevant articles before screening (n = 1035) and facsimiles and extraneous apprenticeships (n= 633), 108 remaining full-text papers were read to assess their suitability and evaluated by the researchers. In addition, systematic studies were examined. After excluding full-text papers as per pre-specified exclusion criteria (n= 46), 62 [1-62] research works were included in this systematic review. A few studies appeared to meet the inclusion criteria but were excluded due to age group and study design. Among 23 studies [8,9,11,13-16,23,28,32,35,36,38,43,51-57,60,61], 22 are randomized controlled trials [8,9,11,13-16,23,28,32,35,36,38,43,51-55,57,60,61], and one study [56] is a pooled analysis from five randomized studies. There were six quasi-experimental trials [17,20,27,31,34,40] and 10 experimental or interventional studies [10,12,19,21,24-26,42,59,62].

In terms of sample sizes, they ranged from 51 to 5926 participants [8-17,19-21,23-28,31,32,34-36,38,40,42,43,51-55,57,59,60-62]. Fourteen studies [11,12,17,24,27,31,34,36,38,40,53,57,59,60] had fewer than 500 participants, 12 studies [8,9,13,20,21,23,25,35,42,43,54,62] had between 501 and 1000 participants, and another 12 studies [10,14-16,19,26,28,32,51,52,55,61] had more than 1000 participants.

Children between the ages of six and 12, and teenagers between 13 and 17 years, were the target groups for this systematic review, as specified in the inclusion and exclusion criteria.

Regarding intervention types, 13 studies involved extensive multi-component strategies [8-16,31,34,52,55], 15 were nutrition education interventions [17,19,20,21,23-27,51,57,59-62], six focused on physical activity interventions [28,32,35,36,38,54], and four on lifestyle and nutrition-related behavior [40, 42, 43, 53]. Another 24 of the 62 included studies [1-7,18,22,26,29,30,33,37,39,41,44-50,58] were original articles, review articles, and government guidelines, protocols, policies, and reports regarding school children and adolescents’ nutritional status.

The duration of the interventions ranged from one day [12] to five years [52], with 25 studies [8,10-12,17,19-21,26-28,32,34-36,38,40,42,43,53,54,57,59,60,62] lasting less than one year. Post-intervention follow-ups, conveyed in 26 research articles [9,11,13,15,17,19,20,23,26-28,31,35,36,37,40,42,43,51-55,57,60-62], ranged between one week [28] and five years [52].

Regarding study settings, all the included studies were conducted in schools or educational institutions, and they had outcomes such as nutritional status, knowledge, attitude, practices, dietary habits, diet pattern, food preferences or choices, nutrition-related knowledge, practice of healthy eating, lifestyle, and behavioral change in relation to nutrition, physical activity, and body composition (BMI, WHO Growth References). Table 3 provides a summary of the key characteristics of the included studies, and Table 4 presents strategies in different combinations in multi-component school programs.

**Table 3:** Characteristics of the included studies. (-) *: Not  mentioned; HHS: Healthy High School.

Author, year and study location	Study setting and design	Intervention	Participants and age during intervention	Sample size	Duration of the intervention	Follow up and number of dropouts	Outcomes
Fonseca LG et al., (2019) [8], Brasilia, Federal district, Brazil	School, Randomized Controlled Trial	Comprehensive problem-raising approach and pictorial representations	Brazilian adolescents, Mean age (years) =14.8±1.0	n=676	4 months	0 months, n=215	Improved knowledge and practice of healthy eating
Scherr RE et al., (2017) [9], California	School (Randomized Controlled Trial)	Multicomponent healthy choice program	Upper elementary School children and adolescents (9 -12 years)	n=566	1 year	2 months, n=82	Nutrition knowledge, dietary behavior, BMI
Efthymiou V et al., (2022) [10], Athens, Greece	School, Multi-level intervention study	Comprehensive and multicomponent lifestyle intervention	Adolescents (12-17 years)	n=1610	6 months	0 months, n=590	Diet and exercise, BMI, Waist-Circumference, Waist to Height ratio, Waist to Hip ratio
Leme AC et al., (2018) [11], São Paulo, Brazil	School (Randomized Controlled Trial)	Multicomponent intervention	Girl school children (15.6±0.87 years)	n=253	7 months	6 months, n=109	Height and weight, waist circumference, dietary behavior
Raikar K et al., (2020) [12], Delhi, India	School, Intervention study	Comprehensive nutrition education using flip chart and two way discussion	School going adolescent girls from class 9th standard (13-15 years)	n=286	1 day	0 months, n=21	Nutrition knowledge
Ofosu NN , et al., (2018) [13], Canada	School, Randomized Controlled trial	Kids’ Health research project	School children and adolescents (10-15 years)	n=540	1 year	1 year, n=59	Health-related knowledge, attitudes, dietary intake, activity and weight status
Xu H et al., (2020) [14], China	School, Randomized Controlled Trial	Comprehensive nutrition education and physical activity intervention	School children (7-13 years)	n=4846	1 year	0 months, n=87	Overall dietary diversity, dietary habits, nutrition related knowledge, BMI
Duus KS et al., (2022) [15], Denmark	School, Cluster Randomized Controlled Trial	Healthy High School (HHS) multicomponent Intervention on lifestyle	School students (6-7 years and 15-16 years)	n=4512	1 year	9 months, n=65	Healthy dietary habits, meal frequency
Liu Z et al., (2022) [16], China	School, Cluster Randomized Controlled trial	Multi-faced obesity intervention	School children (8-10 years)	n=1392	1 year	(-)*	BMI, physical activity, dietary behavior, obesity related knowledge
Glorioso IG et al., (2020) [17], Laguna, Philippines	School, Quasi experimental	Nutrition education program for healthier kids	Grade II and III students (7-9 years)	n=166	8 months	Total 5 follow-up meetings), n=0	Improved knowledge on food and nutrition
Hamulka J et al., (2018) [19], Poland	School, The study was designed in two paths as: a cross-sectional study and an intervention study	Health educational intervention	Girls aged 11-13 years	n=2107	3 weeks	9 months, n=74	Nutrition and lifestyle related knowledge, dietary and lifestyle behaviors and body composition
Teo CH et al., (2021) [20], Malaysia	School, Quasi experimental study	Educational intervention	School children (7-11 years)	n=523	3 months	3 months, n=0	Anthropometric assessments, nutrition knowledge and eating practice
Anand D et al., (2020) [21], Tirupati, India	School, Intervention Study	Health and nutrition education program	Adolescent girls aged 13-17 years	n=700	6 weeks	0 months, n=0	Dietary pattern, anthropometric assessment, nutritional status
He FJ et al., (2022) [23], China	School, Cluster randomized Controlled trial	Nutrition education program	School children (8-9 years)	n=592	1 year	3 months, n=27	Dietary habits, practices, nutritional status
Aydın S et al., (2022) [24], Usküdar, Istanbul	School, Experimental or Intervention study	Nutrition education	School students (14-17 years)	n=216	(-)*	0 months, n=0	Nutrition related knowledge, BMI, dietary habits and lifestyle
Hildrey R et al., (2021) [25], Storrs, USA	School, Intervention study	Tailored Nutrition Education	School children from 6th, 7th, 8th standard (10-15 years)	n=505	1 year	(-)*, n=0	Nutrition related knowledge, behavior in dietary habits, physical activity
Seneviratne SN et al., (2021) [26], Colombo, Sri Lanka	School, Pre-post study design, Intervention study	Nutrition education	School children from grade 1 and 2	n=1042	1 week	At the 2nd week post-test, n=0	Eating habits, nutritional status
El Bastawi S et al., (2022) [27], Mansura, Egypt	School, Quasi-Experimental study	Educational intervention Program	School Children (7-12 years)	n=360	1 week	1 month, n=5	Nutrition related knowledge, attitude, dietary habit
Habib-Mourad C et al., (2020) [28], Lebanon	School, Randomized Controlled Trial	Physical activity intervention	School students (9-11 years)	n=2148	3 months	1 week, n=128	Dietary behavior, Nutrition knowledge; physical activity
Seo YG et al., (2021) [31], Korea	School, Quasi-experimental	Circuit training and nutrition intervention	School children (6-17 years)	n=242	2 years	6 months, n=79	BMI, body composition, nutrition and physical activity
Larsen MN et al., (2021) [32], Denmark	School, Cluster Randomized Controlled Trial	Health Education through football program	School children (10-12 years)	n=3127	11 week	0 months, n=3005	Hygiene, nutrition, physical activity and well-being
Tapia-Serrano MA et al., (2022) [34], Spain	School, Quasi-Experimental design	Physical activity, lifestyle and dietary intervention	School children (8-9 years)	n=121	2.5 months	0 months, n=0	Self-rated health status, BMI, physical activity, sleep duration, dietary habit and sedentary screen time
Martínez‐Vizcaíno V et al., (2022) [35], Cuenca, Spain	School, Cluster Randomized Controlled Trial	Physical activity intervention	School children (9-11 years)	n=562	6 months	1 academic year, n=166	Changes in physical fitness parameters, body composition (BMI, waist circumference) blood pressure, and biochemical parameters
Sebire SJ et al., (2018) [36],	School, Cluster Randomized Controlled trial	Physical Activity Intervention	Adolescent girls (12-13 years)	n=427	6 months	2 months, n=39	Moderate to vigorous physical activity and sedentary time
Santina T et al., (2021) [38], Lebanon	School, Cluster randomized Controlled trial	Physical Activity Intervention	School Children (10-12 years)	n=374	14 weeks	0 months, n=10	Physical activity, behavior in relation to dietary habit, BMI, waist circumference
Sharif Ishak SI et al., (2020) [40], Malaysia	School (Quasi-experimental)	lifestyle program	Adolescents (13-14 years)	n=201	16 weeks	3 months, n=125	Knowledge, attitude and practice on healthy lifestyle and anthropometric measurements
Kamin T et al., (2022) [42],	School, Experimental	Behavior change in connection with dietary habits	Schoolchildren (10-16 years)	n=672	5 months	4 months, n=131	BMI, dietary habit, behavior change in terms of dietary habits, awareness of health risks related to consumption of sugar sweetened beverages
Moitra P et al., (2021) [43], Mumbai, India	School, Cluster randomized Controlled trial	Behaviorally focused nutrition education intervention	Students (10-12 years)	n=518	12 weeks	2 months, n=20	Knowledge, attitude, practice and diet
Ochoa-Avilés A et al., (2017) [51], Cuenca, Ecuador	School, Cluster randomized control trial	Nutrition education	Adolescents 12-14 years	n=1079	2 years and 4 months	1st follow up 17 months, 2nd follow up 11 months	Healthy dietary habits, physical activity, BMI, reduced waist circumference
Lane HG et al., (2017) [52], Maryland	School, Cluster randomized control trial	Multi-component intervention	School children from 3rd to 7th grade (6-12 years)	n=1080	5 years	2.5 years, n=0	Obesogenic behavior and weight, BMI, physical activity, health literacy
Kubik MY et al., (2018) [53], Philadelphia, United States	School, Randomized Controlled Trial	Lifestyle intervention	School children 8-12 years	n=114	9 month	At 12 months post intervention and 24 months follow up	Child age- and gender-adjusted BMI z-score, dietary intake, physical activity
Ten Hoor GA et al., (2018) [54], The Netherlands	School, Cluster Randomized Controlled Trial	Physical activity intervention	Adolescents 11-15 years	n=695	6 months	At 12 months, n=187	Body composition, daily physical activity, sedentary behavior
Silva KS et al., (2020) [55], Brazil	School, Cluster Randomized Controlled Trial	Multi-component intervention	Adolescents 7th to 9th grade school children	n=1090	1 year	2 months n=91	Physical activity, Sedentary behavior, self-efficacy, nutritional status
Ponnambalam S et al., (2022) [57], Puducherry, India	School, Randomized Controlled Trial	Nutrition education	Adolescents aged 11-14 years	n=280	9 months	9 months n=0	Waist circumference, eating behaviors
Haney MO et al., (2017) [59], Turkey	School, Intervention study	Educational Intervention	School children from 4th grade	n=51	2 weeks	(-)*	Dietary habits, BMI
Bagherniya M et al., (2017) [60], Iran	School, Cluster Randomized Controlled Trial	Nutrition education intervention	Adolescent overweight girls aged 12-16 years	n=172	7 months	At 3.5 months and 7 months, n=18	BMI, Waist circumference, dietary intake and behavior
Habib-Mourad C et al., (2020) [61], Beirut, Lebanon	School, Cluster Randomized Controlled Trial	Nutrition education intervention	School children from 4th -5th grade (8-12 years)	n=1239	2 years	1 year, n=433	Dietary habits, Physical activity, knowledge, Self-efficacy
Wadolowska L et al., (2019) [62], Poland	School, Intervention study	Nutrition education intervention	School children aged 11-12 years	n=668	9 months	At 9th month, n=187	BMI, Waist to Height Ratio, Diet quality, Nutrition Knowledge, sedentary and active lifestyle

**Table 4:** Strategies in different combinations in multi-constituent school programs.

Multi-constituent strategies
Comprehensive nutrition intervention strategies
Nutrition education
Diet counselling
Lifestyle intervention
Behavioural intervention related to dietary habits
Physical activity intervention
School health promotion program

Discussion

This systematic review is an effort to bring together various evidence-based studies on effective school-based nutrition interventions that promote healthy dietary practices, food preferences, lifestyle, diet-related behavior, and knowledge, as well as the nutritional status of school children and adolescents. It has been found that health and nutrition-related intervention programs significantly impact the health, knowledge, lifestyle, and behavioral status of school-aged children and adolescents in relation to dietary habits. This underscores the need to increase mindfulness about the benefits of including whole foods in daily diets [7,8,10,11]. Of the examined studies, 34 out of 38 demonstrated favorable correlations between specific interventions and improved outcomes [8-17,19-21,23-28,31,32,34-36,38,40,42,43,51-55,57,59,60,61,62]. The key interventions were grouped into four categories based on the nature and type of interventions.

Type of Interventions and Their Combinations

Multi-component interventions: Among the evaluated studies, 13 were comprehensive multi-component interventions [8-16,31,34,52,55]. These trials impacted BMI, understanding of nutrition, practice of healthy eating, dietary diversity, level of physical activity, self-efficacy for exercise, waist circumference, and personal hygiene. Nutrition and health education using a problem-raising approach, utilization of visual aids like flip charts and picture representations, and lifestyle and behavioral intervention in combination are consistent improvement characteristics across trials [8,9,11,12,14]. These studies also show that health education based on an interactive, participative, problem-raising strategy with the use of food illustrations, combined with physical exercise, can affect dietary intake.

Nutrition Education

Among the evaluated studies, 15 focused on nutrition education interventions [17,19-21,23-27,51,57,59-62]. These studies were classified according to their direct focus on nutrition education. One common factor for improvement was a structured nutrition education program with interactive discussion, practical nutrition lessons, and a dedicated time slot led by professional, trained school teachers and trained peers [15,18,21,24,52]. The impact of these activities is mostly a change in dietary habits and nutrition-related knowledge, justifying the success of these components. The educational activities in the classroom are based on formal education, employing organized material with precise objectives to transmit knowledge about the benefits of eating right.

Physical Activity Interventions

A rise in physical activity and a decline in sedentary behaviors have been identified in six studies [28,32,35,36,38,54]. A self-sustaining, school-based physical activity program run by experts and qualified school teachers with strong nutritional knowledge has been found to be one of the common improvement factors across these studies [28,32,35,36,38,54]. This underlines the importance of implementing interventions that not only concentrate on enhancing a single healthy habit but also design dietary strategies aimed at adopting an overall healthy lifestyle, including physical activity. In this regard, current research shows that addressing two health behaviors simultaneously with a multidisciplinary approach may have an indirect or synergistic impact [14,45,48]. BMI and waist circumference were found to have changed in 25 of the analyzed studies [8,9,12,14,17,18,20,21,25,26,28-30,32,34,35,44-48,50,53-55] after implementing a physical activity intervention program. Four of these studies [10,49,51,52], which used self-report methods to assess changes in health behaviors, revealed no significant changes in BMI and waist circumference. They also had a low retention rate at follow-up, failed to account for a significant number of trials, tracked weight using BMI and waist circumference restrictions, which made it impossible to determine the best possible outcomes, and had a strained intervention that might not be generalizable to other populations. This highlights the need for thorough follow-up, standardized testing, generalizable intervention programs, and the consideration of socioeconomic status in achieving the best outcomes.

Lifestyle and Nutrition-related Behavior Intervention

Lifestyle and behavioral interventions, such as altering eating habits, eating behaviors, and awareness of health risks, significantly improved nutrition-related knowledge, attitude, and practice in healthy lifestyles among school-aged children and adolescents, as demonstrated by four studies [40,42,43,53]. A systematic and well-organized lifestyle intervention program was attributed in one research noted by Sharif Ishak SI et al., 2020 [40] to a decrease in abdominal obesity; this conclusion is also supported by the findings of studies conducted by Kamin T et al., 2022 [42]. These findings imply that lifestyle programs that do not just concentrate on diet are more successful [14-16].

Other Key Findings

**Figure 3:**
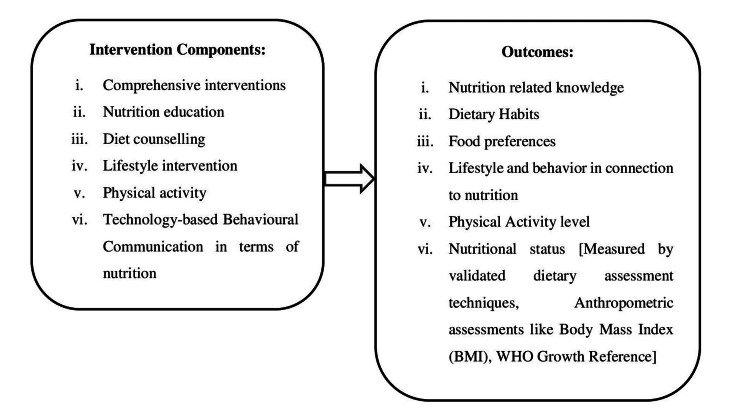
Relationship between intervention components and outcomes.

Regarding the primary attributes of the findings, the sample size ranged from 51 to 5926 [8-17,19-21,23-27,28,31,32,34-36,38,40,42,43,51-54,55,57,59,60,61,62]. The majority of the studies used large sample sizes, specifically between 501 and 2000 participants. Only 14 of the selected studies [11,12,17,24,27,31,34,36,38,40,53,57,59,60] involved fewer than 500 attendees, specifically, ranging from 51 to 500, allowing for reliable results. However, the sizes of the research samples varied significantly. The limited sample sizes could be attributed to various factors, such as studies carried out with specific gender participants (only female) [11,12,19,21,36,60]; in rural areas, which often have smaller populations [17]; lack of a control group and focusing on a particularly selected group of people [24,27,36,40]; not using probability sampling methods [31]; and selecting schools from a small area [34,38]. Despite some research involving a significant number of selected schools, the proportion of students included in the studies was quite low [17,24,31,34,53,57,59,60], often due to loss of follow-up [40]. Intervention duration is another important key feature that can affect the results of intervention programs. Overall, the short and long-term intervention periods provided in the chosen research varied from one day [12] to five years [52], with 25 studies [8,10-12,17,19-21,26-28,32,34-36,38,40,42,43,53-55,57,59,60,62] lasting less than a year. This indicates that these strategies may prove successful in the long run. For example, consider the research done by Scherr RE et al., 2017 [9], Ofosu NN et al., 2018 [13], Xu H et al., 2020 [14], Duss KS et al., 2022 [15], Liu Z et al., 2022 [16], He FJ et al., 2022 [23], Hildrey R et al., 2021 [25], whose duration is one year; by Seo YG et al., 2021 [31] and by Habib-Mourad C et al., 2020 [61], each lasting two years; by Ochoa-Avilés A et al., 2017 [51], lasting two years and four months; and Lane HG et al., 2017 [52], lasting five years. Considering these investigations were conducted in a school setting, it is safe to infer that the intervention was paused at least during the summer break. It is important to highlight in the common findings that the effects of interventions tend to diminish over time [8,10,12,14,21,24,25,32,34,38]. In this respect, it might be assessed to see if the newly learned healthy behaviors are maintained over time, especially after the intervention is complete, as this is the major goal of any research. A recent study that looked at the effect over time of an educational intervention on diet, physical activity, and BMI in children, including adolescents, discovered that the intervention was still effective after two years [31]. In one multi-component nutrition intervention research on BMI by Scherr RE et al. in 2017, significant results were shown one year after the intervention [9]. Improvements in weight were seen in both investigations. There was consistency in the approach used to evaluate participants' nutrition knowledge through the use of various questionnaires [8,9,12,14,16,17,20,24,25,27,28,36,40,43,51-57,59,61,62]. Every type of questionnaire used with children and adolescents follows a proven approach. Although there are several types and proven models for each, there is no consensus on the best method to assess nutritional knowledge. Using questionnaires has drawbacks, as the information gathered may be skewed, among other issues. However, studies have demonstrated that school-age children and teenagers, even as young as six years old, are capable of responding to questions about themselves, including those about their health and nutrition [31]. Figure 3 shows the relationship between intervention components and outcomes.

Risk of Bias assessment 

**Figure 4:**
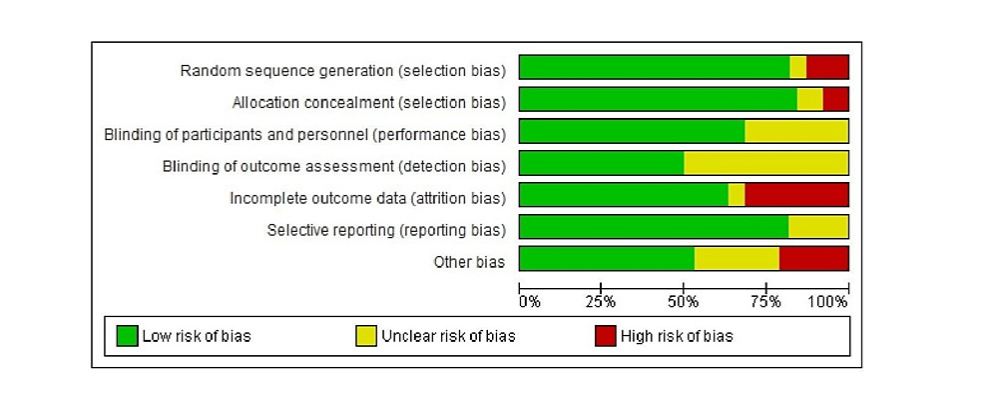
Risk of bias graph about each risk of bias item presented as percentages across the included studies.

**Figure 5:**
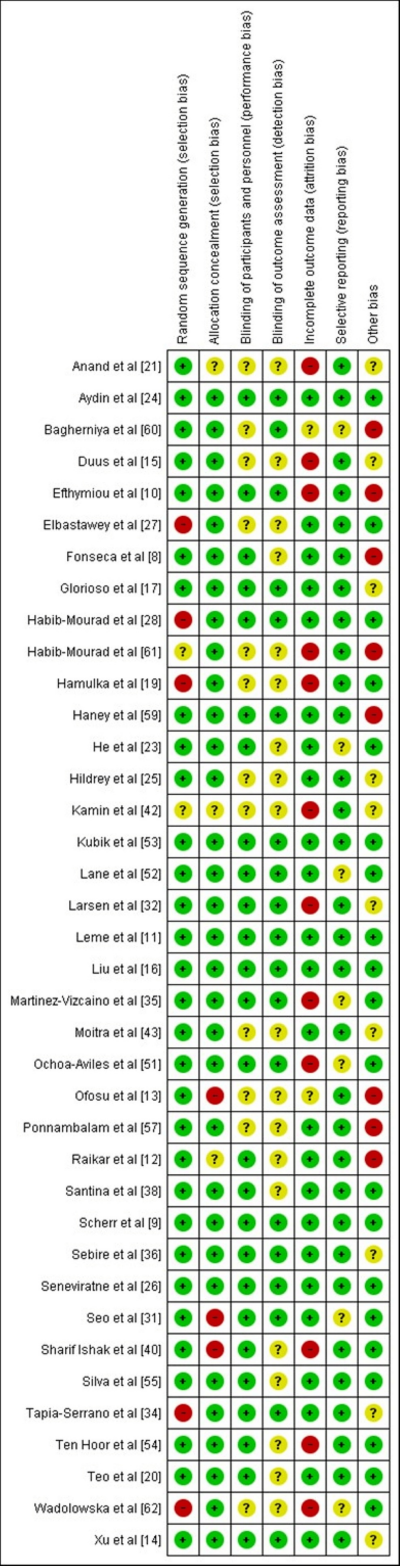
Risk of bias summary of each risk of bias item for included studies. Green: Low risk of bias; Red: High risk of bias; Yellow: Unclear risk of bias.

The Risk of Bias (RoB) assessment has been conducted using Review Manager version 5.4. Figures 4 and 5 illustrate the risk of bias graph and the risk of bias summary of the included studies, respectively. The RoB assessment aims to assess the risk of bias for each of the seven domains in the RoB1 tool. These domains include bias arising from inadequate generation of a randomized sequence; bias due to inadequate concealment of allocations prior to assignment; bias due to knowledge of the allocated interventions by participants and personnel during the study; bias due to knowledge of allocated interventions by outcome assessors; bias due to the amount, nature, and handling of incomplete outcome data; bias due to selective outcome reporting; and any other bias not mentioned above. The signaling questions 'bias arising from inadequate generation of a randomized sequence' and 'bias due to inadequate concealment of allocations prior to assignment' were most often identified. This was in part because studies did not clearly report how the allocation sequence was generated and concealed. Other biases amongst the studies are due to the measurement of outcomes, such as self-reported dietary habits, physical activity outcomes, recall bias, social desirability bias, and others [14,15,17,21,25,32,34,36,42,61]. Furthermore, a few studies failed to explain their sample size estimation [14,17,25,34,36,42]. The lack of information reported in studies regarding certain domains might have masked underlying biases that could not be identified.

Advantages and Limitations

The key advantage of our review is the systematic search strategy performed, which makes it less likely to overlook important regional studies. It also offers many other advantages. For example, this review provides a thorough and current assessment of this subject; it analyzes and assesses each of the factors that can contribute to the success of these techniques; it notes the presence of follow-up sessions, along with the size of the sample and the length of the intervention in the studies that were chosen; it demonstrates the reliability of the data, has used three databases (PubMed, SCOPUS, WOS), and includes 62 related articles. Additionally, this systematic review has a few limitations. Firstly, most findings are based on self-reported data and, hence, have biases such as recall bias, selection bias, and social desirability bias. Secondly, as some studies implemented a multi-interventional program, it is difficult to determine the extent to which reductions in anthropometric markers, e.g., BMI and waist circumference, were due to the physical activity intervention as distinct from other components (such as dietary habit change). Thirdly, further research will be required to determine the relative effects of physical activity interventions alone, both quantitative and qualitative, or a combination, when compared to those of nutrition interventions alone or in combination with each other.

## Conclusions

This systematic review has found that improving both short- and long-term eating behaviors in schoolchildren and adolescents may be accomplished through interventions that alter their environments. However, it is also apparent that the majority of research have not concentrated on various contextual factors, which may have a significant impact on the efficacy of various nutritional interventions. To improve young adolescents' and school-age children's overall health and nutritional status, it is crucial to concentrate on and plan cost-effective, thorough, multi-strategic intervention studies. Exploring strategies that incorporate each of the components stated below might be intriguing to have a greater impact on the nutrition and health of schoolchildren and the teenage population: (a) approaches that emphasize acquiring a healthy lifestyle overall rather than only changing eating practices, including physical exercise as part of the intervention; (b) due to the significant influence parents have on their children's eating habits, interventions that focused on the school and the settings of children engaged parents in the intervention; (c) techniques that incorporate health knowledge, such as classes on nutrition; and (d) techniques that assess long-term impacts after an intervention is complete to see if the positive outcomes are sustained over time. However, it would be intriguing to examine the potential implications of these approaches on other parameters, like changes of body mass and constituents or biomarkers.

Ideally, this review should assist community stakeholders, such as school administrators, teachers, students, parents, policy makers, health professionals, health educators, and research materials in framing the National Nutrition Education Programs. Such programs address the unfinished agenda of interventions needed to address the dual burden of malnutrition among schoolchildren and adolescents for improving the health status of the community as a whole. Even though school-based nutrition programs have evidence suggesting they can in theory be effective, evidence of the long-term sustainability of these programs has yet to be studied or reviewed in any formidable detail.

## Appendices

Appendix 1

Literature Selection Strategy

Electronic databases have been used to enable a thorough, systematic investigation into research like PubMed/ Medline, SCOPUS, WOS and was searched until 2023 July to identify relevant studies. Exploration was restricted to journals with tutelages available in English and English titles, abstracts and keywords with phrases such-as “body mass index/BMI”, “Underweight/ under-nutrition”, “Obesity”, “Malnutrition”, “School”, “Lifestyle”, “Dietary habits”, “Food choices”, “Physical activity”, “Personal hygiene”, “Children”, “Adolescent”, “Risk factors”, “Intervention”, “Strategies”, etc.

PubMed search is standardized appropriately: (school) AND ((physical activity) AND (physical education) AND (exercise) AND (physical fitness) AND (sports) AND (lifestyle) AND (nutrition) AND (malnutrition) AND (intervention) AND (nutrition education) AND (energy intake) AND (energy density) OR (calories) AND (food) OR (fruit) OR (vegetable)) AND ((weight) OR (obese) OR (overweight) OR (weight reduction) OR (under-nutrition) OR (anthropometric) OR (anthropometry) OR (nutritional status) AND (nutrition assessment) OR (BMI) OR (waist circumference) OR (adipose tissue)) AND (child[MeSH:noexp] OR adolescent[MeSH])).

SCOPUS search is standardized appropriately: (KEY(School based) AND TITLE-ABS-KEY(physical education) AND TITLE-ABS-KEY(physical exercise) AND NOT TITLE-ABS-KEY(Sports) AND TITLE-ABS-KEY(Nutrition intervention, nutrition education) AND NOT TITLE-ABS-KEY(Supplementary nutrition, meal consumption) AND TITLE-ABS-KEY(Food preferences, dietary habits) AND TITLE-ABS-KEY(Lifestyle, behavior) AND TITLE-ABS-KEY(Body weight) AND TITLE-ABS-KEY(Obese) AND TITLE-ABS-KEY(Overweight) AND TITLE-ABS-KEY(Body Mass index) AND TITLE-ABS-KEY(Waist circumference) AND TITLE-ABS-KEY(School children) AND TITLE-ABS-KEY(Adolescents)) AND PUBYEAR > 2016 AND PUBYEAR < 2024 AND ( LIMIT- TO (LANGUAGE, English)).

Web of Science (WOS) search is standardized appropriately: nutrition intervention (Topic) and school (All Fields) and children (All Fields) and adolescents (All Fields) and habits (All Fields) and dietary (All Fields).
